# Treatment of Residual Periodontal Pockets Using a Hyaluronic Acid-Based Gel: A 12 Month Multicenter Randomized Triple-Blinded Clinical Trial

**DOI:** 10.3390/antibiotics10080924

**Published:** 2021-07-30

**Authors:** Andrea Pilloni, Blerina Zeza, Davor Kuis, Domagoj Vrazic, Tomislav Domic, Iwona Olszewska-Czyz, Christina Popova, Kamen Kotsilkov, Elena Firkova, Yana Dermendzieva, Angelina Tasheva, Germano Orrù, Anton Sculean, Jelena Prpić

**Affiliations:** 1 Department of Dental and Maxillo-Facial Sciences, Section of Periodontology, Sapienza University of Rome, 00161 Rome, Italy; andrea.pilloni@uniroma1.it; 2 Department of Periodontology, School of Dental Medicine, University of Rijeka, 51000 Rijeka, Croatia; kuis@net.hr (D.K.); jelena.prpic@fdmri.uniri.hr (J.P.); 3 Department of Periodontology, University of Zagreb, 10000 Zagreb, Croatia; vrazic@sfzg.hr; 4 Dental Polyclinic Zagreb, Perkovceva 3, 10000 Zagreb, Croatia; tomislavdomic@yahoo.com; 5 Department of Periodontology, Jagiellonian University Medical College, ul.Montelupich 4, 31-155 Krakow, Poland; iwona.olszewska-czyz@uj.edu.pl; 6 Department of Periodontology, Faculty of Dental Medicine, Medical University of Sofia, 1431 Sofia, Bulgaria; hristina_popova@fdm.mu-sofia.bg (C.P.); kamenkotsilkov@gmail.com (K.K.); 7 Department of Periodontology, Faculty of Dental Medicine, Medical University of Plovdiv, 4004 Plovdiv, Bulgaria; Elena.Firkova@mu-plovdiv.bg (E.F.); yana.dermendzieva@mu-plovdiv.bg (Y.D.); p.v.tashev@gmail.com (A.T.); 8 Oral Biotechnology Laboratory (OBL), Department of Surgical Sciences, University of Cagliari, 09124 Cagliari, Italy; gerorru@gmail.com; 9 Department of Periodontology, School of Dental Medicine, University of Bern, 3010 Bern, Switzerland; anton.sculean@zkm.unibe.ch

**Keywords:** hyaluronic acid, periodontitis, periodontal therapy, residual pockets

## Abstract

The aim of the present study was to evaluate the adjunctive effect of hyaluronic acid (HA) gel in the treatment of residual periodontal pockets over a 12-month period. Periodontal patients presenting at least one residual periodontal pocket 5–9 mm of depth in the anterior area were recruited from six university-based centers. Each patient was randomly assigned to subgingival instrumentation (SI) with the local adjunctive use of HA for test treatment or adjunctive use of local placebo for control treatment at baseline and after 3 months. Clinical parameters ( )probing depth (PD), bleeding on probing (BoP), plaque index (PI), recession (REC), clinical attachment level (CAL)) and microbiological samples for the investigation of the total bacterial count (TBC) and presence of specific bacterial species (*Porphyromonas gingivalis*, *Treponema denticola*, *Tannerella forsythia*, *Fusobacterium nucleatum*) were taken at baseline and every 3 months, until study termination. PD was determined as the primary outcome variable. From a total of 144 enrolled, 126 participants (53 males, 73 females) completed the entire protocol. Both treatments resulted in statistically significant clinical and microbiological improvements compared to baseline. Although the local application of HA showed a tendency for better results, there was a lack of statistically significant differences between the groups.

## 1. Introduction

Periodontitis is still among the most globally prevalent oral diseases, despite the observed improvement during recent decades in countries with high incomes [1,2,3].

If treated, disease progression can be stopped [4]. However, only patients with residual periodontal pockets ≤4 mm after active periodontal therapy are more likely to present stability of clinical attachment level over a follow-up time of beyond 1 year [5,6]. The goals of periodontal therapy and subsequent maintenance should be to reduce or eliminate residual probing depths which are considered optimal ecological niches for the pathogenic microbiota whilst keeping the resistance and resilience of the patient at a high level [7].

In periodontitis patients with deep probing depths (≥6 mm) or complex anatomical surfaces (root concavities, furcations, infrabony pockets) Step 1 (adequate patient’s oral hygiene practices, professional elimination of supragingival biofilm and risk factor control) and Step 2 (elimination/reduction of supra and subgingival biofilm and calculus, with or without adjunctive therapies) may not lead to the achievement of the endpoints of therapy, and further treatment should be implemented, such as repeated subgingival instrumentation (SI) with or without adjunctive therapies or periodontal surgery [8].

Hyaluronic acid (HA) has been added to the local chemotherapeutic agents due to its properties in promoting wound healing and periodontal regeneration [9,10,11,12]. In vitro studies confirmed that commercially available high-molecular-weight (MW) HA products are highly biocompatible and (a) in gingival tissues do not impair the healing process by prolonging inflammation or causing excessive MMP expression at the repaired site [13]. When applied in the dentin surface, (b) it increases the dentin surface roughness/texture with a subsequent increase in the number and improvement in the spreading of human periodontal ligament cells (PDL) on this surface [14]; (c) maintains high human periodontal ligament (PDL) cell viability, increased proliferation, and early osteogenic differentiation but significantly decreases the late osteogenic differentiation of primary human PDL cells [15,16]; (d) strongly induces the growth of osteoprogenitors and maintains their stemness, thus potentially regulating the balance between self-renewal and differentiation during bone regeneration following reconstructive oral surgeries [17].

The adjunctive application of HA on the surgical treatment of periodontal bone defects and mucogingival defects has shown promising results [12,18,19,20,21]. When applied as monotherapy or as adjunct to non-surgical periodontal therapy based on the aforementioned properties in promoting healing and regeneration, it has shown a positive, occasionally statistically significant, albeit generally moderate effect, on clinical parameters in favor of HA application [10,22]. However, no evidence could be found on the effect that HA could have on residual pockets.

The objective of this study was to evaluate the clinical and microbiological efficacy of hyaluronic acid gel in the treatment of residual periodontal pockets over a 12-month period.

## 2. Results

### 2.1. Population Characteristics

A total of 144 participants gave their consent to participate in the study. Only 126 participants (53 males, 73 females) completed the entire protocol with a drop-out of 12.5% due to lack of compliance or systemic health issues not related to the specific treatment performed. No patient presented any complication during the entire study.

Sixty-four patients (28 males and 36 females; 50.5 ± 10. 7 years old) were randomly allocated to the test group and 62 patients (25 males and 37 females; 50.1 ± 14. 2 years old) were randomly allocated to the control group, where placebo was locally administered to the periodontal pockets.

Both groups were comparable in age and gender distribution and in clinical and microbiological baseline parameters.

### 2.2. Clinical Evaluation

The clinical characteristics of both groups and the differences among them and over time are summarized in Table 1.

**Figure 1:**
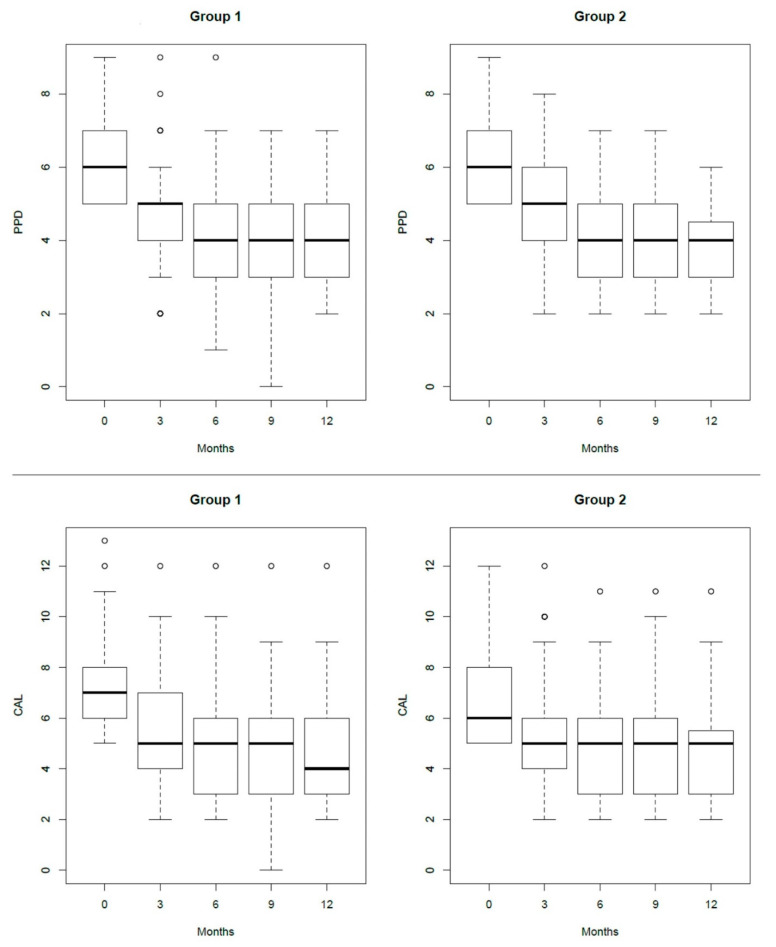
Box plot showing the median, interquartile range, minimum and maximum values (the square and the two extremities) and the outlier values (the circles) of PPD and CAL differences in the same group and between groups.

Step 3 therapy resulted in a significant improvement in time of probing depth (PPD) in both groups (HA: PPD0 = 6 (5–7) mm, PPD12 = 4 (3–5) mm; *p* < 0.001 and placebo: PPD0 = 6 (5–7) mm, PPD12 = 4 (3–4.5) mm; *p* < 0.001) (Figure 1 and Table 1). This decrease in PPD was associated with the decrease in CAL values (HA: CAL0 = 7 (6–8) mm, CAL12 = 4 (3–6) mm; *p* < 0.001 and placebo: CAL0 = 6 (5–8) mm, CAL12 = 5 (3–5.5) mm; *p* < 0.001) (Figure 1). However, in neither time points did the two adjunctive treatments give significantly different results from each other (Table 1). When PPD was evaluated as a reduction from one recall to the subsequent one and overall reduction at 12 months from baseline, no statistical differences were observed among treatments and among centers.

Overall, the presence of residual pockets was reduced without any statistical differences between groups from 3 months to 12 months, 22.6 to 14.8% for HA group vs. 29.7 to 7.9% for the placebo group. In both groups, the reduction from one time point to the other was not significant.

The percentage of residual pockets conversed in shallow pockets (PPD ≤ 4 mm) improved significantly in both groups with no statistically significant difference between, despite the tendency of higher values in the test group (Table 2). In both groups, the second application of adjunctive treatment did not result in significant improvement.

The percentage of patients achieving the endpoint of therapy (PPD ≤ 4 mm and absence of BOP) increased in time in both groups with no statistically significant difference between them (Table 2). However, the application of HA as adjunct to SI tended to give better results sooner and the results obtained after the first and second administration were almost stable in time.

When residual pockets at baseline were divided in moderate (4–5 mm) and deep (6 mm and more), the HA group had a prevalence of 73% (45/62) deep pockets, whereas the placebo group had a prevalence of 66% (42/64) deep pockets. The complete closure of the pocket (PPD ≤ 3 mm) presented different features when the pockets were analyzed separately as moderate (4–5 mm) and deep (6 mm and more), though it did not reach statistical difference in any of the recalls. The greater results of the HA group compared to the placebo group were obtained for moderate pockets at 3-month recall (HA 53% vs. placebo 27%), though not reaching statistical difference. Deep pockets at T6 (3 months visit after the second application) reached a statistical difference (*p* value = 0.02). However, overall, only a few deep pockets could benefit from both therapies (HA 13% vs. Placebo 19%). The second application of HA did not show important improvement except for the aforementioned re-evaluation at T6 of deep pockets.

Bleeding on probing expressed as the percentage of patients presenting BOP at the treated site improved significantly, decreasing in both groups (HA: BOP0 = 77.4%, BOP12 = 37.7%; *p* < 0.001 and placebo: BOP0 = 67.2%, BOP12 = 23.8%; *p* < 0.001), although it did not resolve completely. Both groups were comparable at all time points.

The percentage of patients with plaque at the treated site was reduced in both groups without significant difference (HA: PS0 = 41.9%, PS12 = 26.2%; *p* = 0.7 and placebo:PS0 = 28.1%, PS12 = 21.9%; *p* = 1.0), but the reduction was not stably maintained over time (Table 1).

### 2.3. Microbiological Evaluation

**Figure 2:**
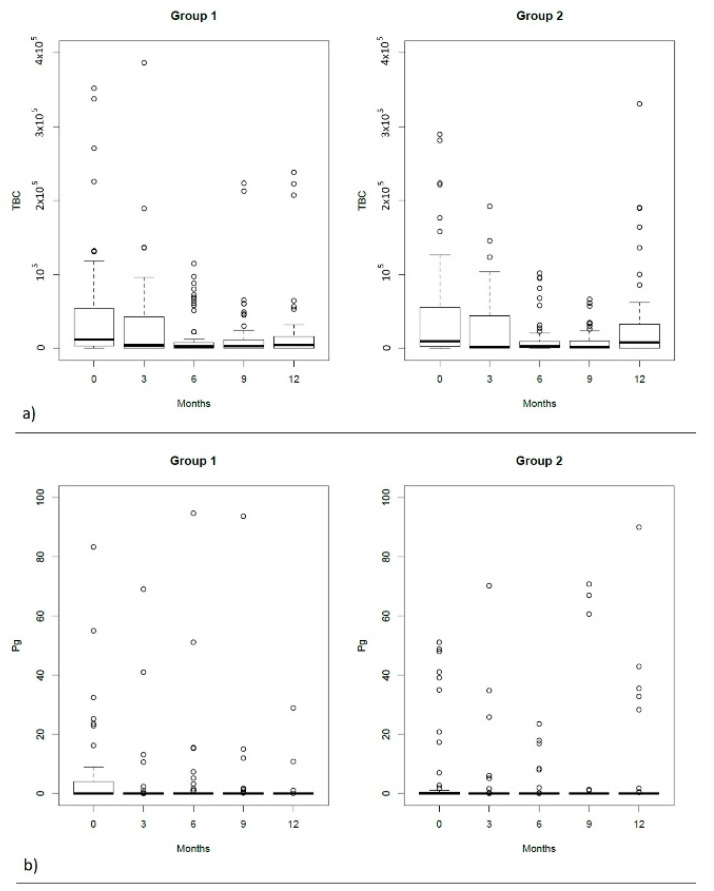

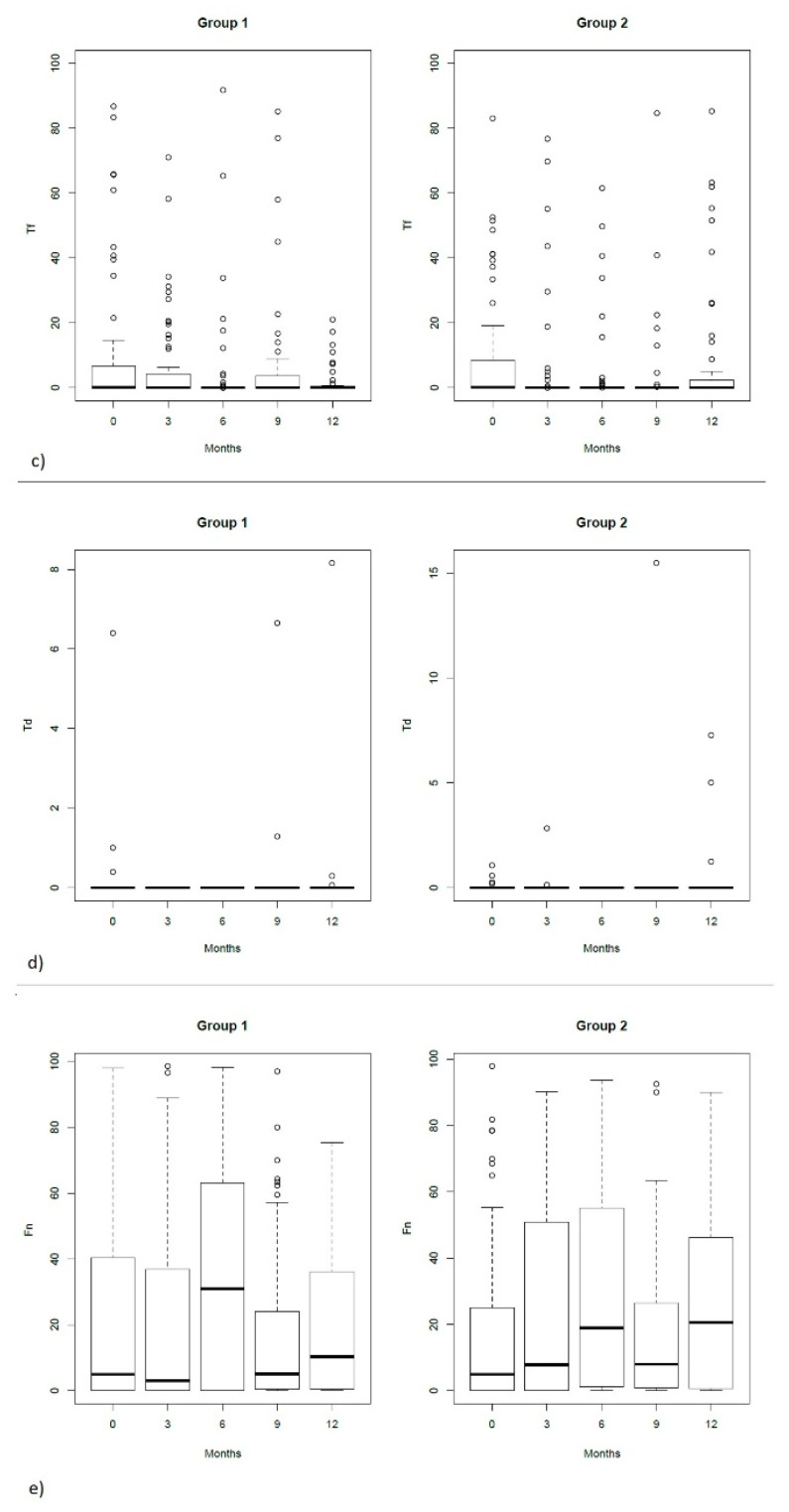
Box plot of the microbiological results. (**a**–**e**) The median, interquartile range, minimum and maximum values (the square and the two extremities) and the outlier values (the circles).

The microbiological characteristics of both groups and differences among them and over time are summarized in Table 3 and Figure 2. The total bacterial count was comparable in all recalls. In both groups there was no evidence of *Treponema denticola*.

In the test group, the total bacterial count (TBC) presented a continuous reduction in time until the 9th month recall when a slight increase was observed but the values constantly remained lower than the baseline. The most important decrease occurred at 3 months recall. The differences observed afterwards did not reach statistical significance between consecutive recalls, despite being statistically significant compared to baseline (*p* < 0.05). In the control group, TBC reduction was not stable, interrupted by two increases at 6 months and at 12 months recall. However, the values remained significantly lower than baseline until the 9th month recall (*p* < 0.001).

*Tannerella forsythia* showed a reduction without reaching a statistical difference in both groups. Only at 6 months recall of the test group was there a significant difference compared to baseline (*p* = 0.003). *Fusobacterium nucleatum* showed particular tendencies noticed in both groups; however, no statistically important differences were noticed in time and between groups.

## 3. Discussion

The evidence on the anti-inflammatory, anti-oedematous, osteoinductive and pro-angiogenetic properties of HA have suggested it as a key material for periodontal wound healing during surgical and non-surgical treatments [22]. In the present multicenter study, a high-molecular-weight HA formulation containing 2.0 mg/mL of sodium hyaluronate and 16.0 mg/mL of sodium hyaluronate cross-linked with butanediol digly-cidyl ether (BDDE) was applied at two different time points at 3 months apart and compared with placebo. The patients were followed for one year at 3-month intervals. The adjunctive use of HA with SI in Step 3 therapy showed a tendency to produce better clinical and microbiological results, although not reaching statistical difference. At 3 months from the first application, the percentage of patients that achieved the endpoint of therapy (PPD ≤ 4 mm and BOP absence) was greater but not statistically significant in the HA group and maintained almost stable over time. Based on the evidence of the importance of this endpoint for the stability of the periodontal condition, it would seem that Ha in adjunct to SI in Step 3 therapy increases the possibility of obtaining such a stability although not reaching statistical importance.

As expected, the greatest improvement was noticed at 3 months after the first application [23].

The present treatment protocol included two rounds of application of HA, in residual periodontal pockets evaluated at 6–8 weeks from Step 1 and Step 2 therapy and 3 months after the first application. Similar protocols of multiple local adjunctive applications have been evaluated in the literature [10,22]. Overall, the second application in the present study did not give important additional improvement to the first application, except for the deep pockets reevaluated 3 months after this second application, but not maintained in the entire follow-up.

The results of the present study are in agreement with those of Chauhan et al., 2013, who reported no statistically significant differences for PD and CAL between SRP (scaling and root planing) alone and the local addition of HA or CHX gel after 3 months of re-evaluation [24]. Similarly, Gontiya and Galgali, 2012, showed no statistically important improvements of PD and CAL after 3 months, although the gingival indexes used (Gingival index and Bleeding index) improved significantly after HA application, contrasting to the present study where no differences were observed concerning the number of positive bleeding sites between groups [25]. On the contrary, Eick et al. 2013 found that the reduction in PPD and in the number of pockets PPD ≥ 5 mm was significantly higher after 3 and 6 months for HA local application compared to SRP alone [26].

In the present study, a mean reduction of 1.45 and 2.25 mm was recorded after 3 months from HA application for pockets with an initial probing depth of 5–6 and ≥7 mm, respectively. The reduction was slightly greater but almost comparable with the reduction after nonsurgical periodontal therapy reported by Graziani et al. (1.29 mm for pockets with an initial probing depth of 5–6 to 2.2 mm for deeper pockets) [27]. A further 0.55 and 0.45 mm reduction was recorded at 6 months recall compared to the 3 months recall. This reduction was associated with a reduction of sites with PD ≥ 5 mm at 3 months recall with 48% and 42% in the HA group and placebo group, respectively. At the end of the protocol, only 24% and 25% of sites presented PD ≥ 5 mm for the HA and placebo group, respectively.

When considering moderate and deep pockets separately, the adjunctive application of HA was more useful in the moderate pockets with a complete closure (PPD ≤ 3 mm) at 3 months in more than twice the cases noticed in the placebo group; however, the difference did not reach statistical significance. In the longer follow-up, the difference between groups narrowed. For the deeper pockets, a statistically significant difference could be observed only after the second application of HA (T6). The fact that the deeper pockets represent 73% of the included pockets in the Ha group and 66% of the pockets in the placebo group, with an overall presence of 69% of deep pockets in the included population, could have been a factor that influenced the overall results of the study. This is in accordance with the suggestions of Sanz et al., 2020, for residual deep pockets after Step 1 and Step 2 therapy to be considerate candidates for periodontal surgery, while moderate pockets to be reinstrumented subgingivally with or without adjunctive therapies.

Although not statistically important, the additional effect of HA at 3 months compared to placebo was 0.31 mm for moderate pockets and 0.32 mm for the deep pockets. These results are comparable and, in some cases, better than the available adjunctive therapies for SI listed by Graziani et al., 2017 [27]. The additional reduction in pocket depth after subgingival irrigation with povidone-iodine was found to be 0.28 mm [28]; for the local antimicrobials, it varied from 0.4 [29] to 0.6 mm [30,31], and an additional clinical attachment gain of up to 0.3 mm may be achieved [32,33] for the combination of amoxicillin and metronidazole, as well as an additional probing depth reduction of 0.58 mm and an additional clinical attachment gain of 0.42 mm [34]; finally, for probiotic treatment, 0.18 mm in probing pocket depth reduction in moderate pockets and 0.67 mm in deep pockets [35], and 0.42 mm in clinical attachment gain were the observed figures [27].

Overall, neither treatment resulted in the complete resolution of all residual pockets. The presence of plaque, even on a single tooth, had a negative impact on the clinical outcome in nonsurgical periodontal treatment [36]. In the present study, the selected teeth were located in the anterior area, which are easier to maintain plaque-free when compared to distal areas, but this did not prevent the presence of plaque during the entire follow-up. This could have been a factor influencing the complete resolution in both groups. A limit of the present study that could have also influenced the obtained results was not considering the presence of other residual pockets in the mouth despite the distant location from the tooth included in the study. The number of deepened or residual pockets or a certain proportion of sites with signs of inflammation are not, per se, by themselves, deterministic for the future stability of attachment level or tooth survival, needed for re-treatment, or even needed to improve oral health-related quality of life because, as a multifactor-natured disease, further periodontal destruction is determined by the interaction of multiple patient factors influencing the host response, and during a life span the factors and the interactions may change [7,37,38,39,40,41]. In this context, the lack of evidence on the establishment and healing process of periodontitis factors such as diet, physical activities, and psycho-emotional conditions [8], does represent a limit in all studies, including the present one.

No traces of Td could be detected in both groups during the entire study. Possible explanations for this could be the fact that this periopathogen is not found in all periodontal patients [42] and that the residual pockets have been already treated in Step 1 and 2 within the 48 h treatment protocol that reduced periopathogens and presumably Td particularly [43]. Along with the clinical parameters, a microbiological improvement was noticed as well, but the difference was comparable between groups, in contrast to the reported improvement from Eick et al., 2013 [26]. Indeed, the available evidence showed bacteriostatic effects of HA [44,45,46].

Although the conclusions of the aforementioned studies on the use of HA were compared with the present one, it should be taken into consideration that in the present study only one site was treated and analyzed in order to prevent the loss of information in the mean values, while in the other studies a mean of the total periodontally compromised sites was presented. The type of HA gel used and the protocol of application in Step 3 therapy in the present study and not during Steps 1 and 2 could also influence the comparison.

When EMD was very recently evaluated for the nonsurgical treatment of residual pockets, even though in the study it was concluded that the results obtained were statistically better than SI alone, the percentage of shallow pockets (PPD ≤ 4 mm) obtained by EMD and HA at 6 months was 89% and 76%, respectively; meanwhile, at 12 months it was 80% and 77%, respectively [47]. Concerning the endpoint of therapy (PPD ≤ 4 mm without BOP), at 6 months, EMD and HA could obtain 69% and 58%, respectively, while at 12 months they could obtain 80% and 59%, respectively. The results obtained for the shallow pockets are comparable. However, the operator-dependent nature of the BOP parameter included in the endpoint of therapy, the percentage of deep pockets in the present study (69%), the different population sample and the different study design could be considered factors influencing the results and conclusions between the two studies. Furthermore, it is evidenced that the blood proteins compete with EMD amelogenins’ absorption in the root surface, thus reducing EMD effectiveness, and it has been advised to minimize blood intervention to allow for better absorption of EMD to root surfaces [48]. On the contrary, HA and blood seem to cooperate with each other, as HA stimulates clot formation and inhibits clot lysis along with the stimulation of angiogenesis [49,50].

## 4. Materials and Methods

### 4.1. Population

A total of 144 patients were enrolled from 6 university clinical centers (Sapienza University of Rome, Department of Periodontology, Rome, Italy; University of Rijeka, School of Dental Medicine, Rijeka, Croatia; University of Zagreb, Department of Periodontology, Zagreb, Croatia; Jagiellonian University, Dental Institute, Krakow, Poland; Medical University of Sofia, Faculty of Dentistry, Sofia, Bulgaria; Medical University of Plovdiv, Faculty of Dentistry, Plovdiv, Bulgaria). The study is registered on ClinicalTrial.gov (Reg. no. NCT04702334) and approved by the ethical committees of each center. The ethical principles of the Helsinki Declaration on the good conduct of clinical trials were followed. All patients were thoroughly informed on the treatment details and on the possibility of being excluded from the study if missing any of the 3 months follow-up appointments or showing poor compliance in general. After verbally accepting the conditions of the protocol, patients signed the informed consent prior to study initiation.

The extension in time from patient recruitment to the end of the protocol was January 2016 to November 2018.

The sample size was determined considering the patient level as a statistical unit and probing depth (PD) as the primary outcome measure. In order to detect a mean difference of 1 mm of PD between groups with a standard deviation of 1 mm, maintaining a power of 85%, a significance level (alpha) of 0.05 and a drop-out of 25%, 12 participants per group in each center were required.

In each center, patients with at least 18 years of age, systemically healthy, smoking less than 10 cigarettes/day, have been treated for periodontitis but with at least one single residual pocket of 5–9 mm of depth located in the anterior area (incisors and canines), full-mouth bleeding score (FMBS) and/or full-mouth plaque score (FMPS) less than 20%, and showing a good compliance to oral hygiene instructions were included in the study.

The exclusion criteria applied were pregnancy/lactation, use of antibiotics in the last 3 months for a period of two weeks or more, use of drugs causing gingival hyperplasia, use of corticosteroids or anti-inflammatory drugs for a period of two weeks or more, presence of diseases known to influence the wound healing/tissue response, thyroid or pituitary gland malfunction or abnormal hormonal levels, hyaluronic acid contraindications, subjects under anticoagulant treatment, radiation therapy in the head and neck area, use of bisphosphonate drugs prescribed for patients with osteoarthritis, and tooth mobility ≥1 degree.

### 4.2. Study Design, Randomization, Allocation Concealment and Blinding

**Figure 3:**
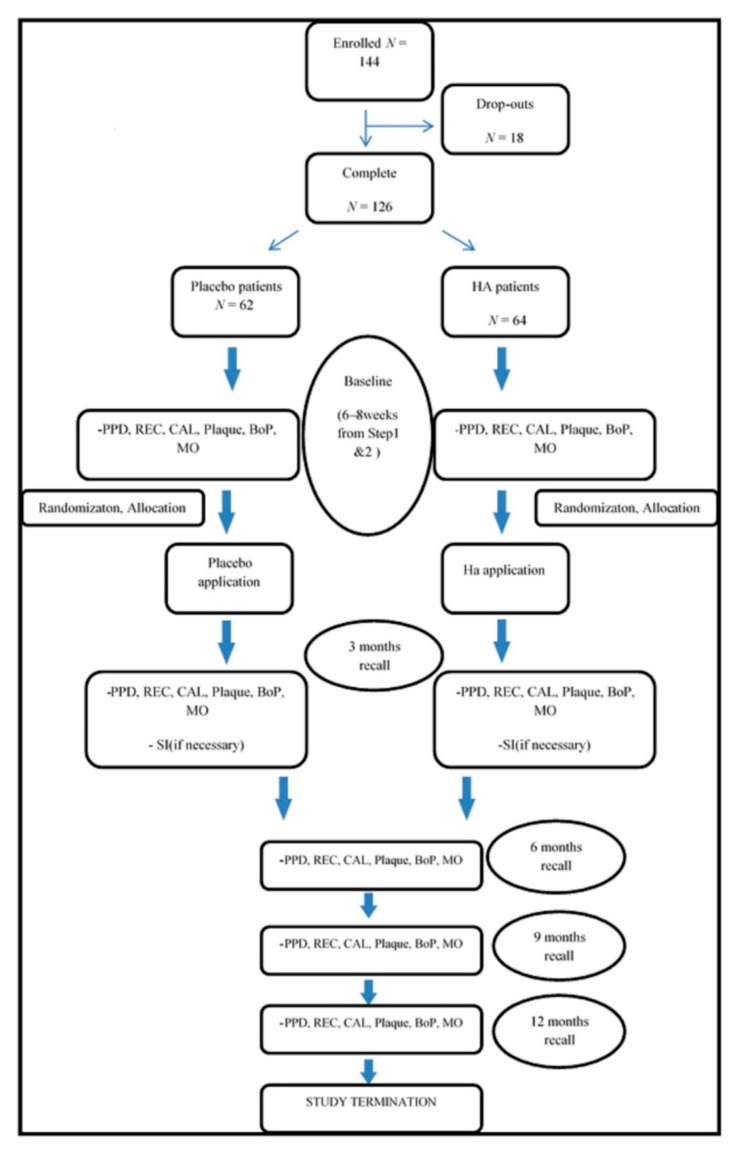
Protocol flow chart.

This study was planned as a controlled randomized multicenter triple-blinded 12-month clinical trial to compare the treatment of residual periodontal pockets using SI with the subgingival application of HA or placebo. Figure 3 shows step by step the study protocol that each patient followed.

The detailed description of the treatment and evaluation is described in the following paragraphs.

The operators were trained and calibrated on treatment application and on PD measurement prior to study initiation. A preliminary session of PD measurements on 3 periodontal patients revealed an intra-class correlation coefficient ≥0.75 for each operator and among different operators.

An electronic web-based online randomization system was used for this study (Institute for Medical Informatics, Statistics and Documentation, Medical University of Graz, Graz, Austria) including a simulation version of the software for operator training prior to trial initiation. The allocation concealment was done by means of an independent computerized randomization sequence process which can only be accessed by an independent trial coordinator. The investigators were provided with an online login and were instructed to perform the randomization only after finishing the baseline measurements and SI of the selected tooth. To ensure this, investigators were requested to write down the exact time on provided case report forms upon finishing debridement. The computerized time stamp, electronic logs of sent allocation, served as a method for monitoring allocation concealment. Furthermore, having the randomization as parallel with stratified (stratification by center) permuted blocks served as a second layer of preventing knowledge of upcoming patient’s assignment.

Clinical operators were kept blinded until adjunctive treatment allocated by the randomization. Patients remained blinded during the entire follow-up. Microbiological evaluation and statistical interpretation of the results were made blinded on the treatment performed.

### 4.3. Treatment Procedure

Each center was provided with the same amount of hyaluronic acid (Hyadent^®^ BG, BioScience GmbH, Dümmer, Germany), Eppendorf tubes, curettes (Hu-Friedy, Chicago, IL, USA), toothpastes (Oral-B Pro-expert, Procter and Gamble, Rome, Italy), toothbrushes (TePe Munhygien produkter AB, Malmö, Sweden) and interdental brushes (TePe Munhygien produkter AB, Malmö, Sweden). In the present study, a high-molecular-weight HA formulation containing 2.0 mg/mL of sodium hyaluronate and 16.0 mg/mL of sodium hyaluronate cross-linked with butanediol digly-cidyl ether (BDDE) was used. Patients were instructed to use the modified Bass technique for brushing twice daily with the provided toothpaste free of chlorhexidine and to use the interdental brush once daily. They were instructed not to use any type of mouthwash.

All patients completed Step 1 and Step 2 therapy within 48 h in two consecutive visits using ultrasonic devices and curettes for all pockets of more than 4 mm. Possible plaque-retentive factors impairing oral hygiene practices were corrected as well.

At re-evaluation (6–8 weeks), a tooth entering the inclusion criteria was selected. After the application of local anesthetic, Step 3 therapy was performed by supra (if necessary) and subgingival instrumentation in addition to the local administration of HA gel for the test group and local administration of anesthetic solution (placebo) for the control group. The tip of the blunt application needle mounted on the syringe containing HA was gently inserted in the pocket until resistance was felt. The injection was performed until the remnants started getting out of the pocket. Patients were advised not to drink or eat for 2 h. This adjunctive treatment was performed at baseline (re-evaluation of Step 1 and 2) and 3 months later. SI was performed only at baseline. After taking the plaque samples and measuring the clinical parameters, in case of the presence of plaque, supragingival removal was performed at the selected tooth. For both groups, cleaning of supragingival tooth deposits and polishing in the entire dentition were repeated at 3, 6, 9 and 12 months.

### 4.4. Treatment Assessment

#### 4.4.1. Clinical Assessment

The patients were evaluated clinically and microbiologically at baseline and at 3, 6, 9, and 12 months after Step 3 therapy. Clinical parameters were measured by a single operator at each center as follows: probing depth (PD) in mm, bleeding on probing (BoP) and plaque measured as a dichotomous parameter (PI), and recession of gingival tissues (REC). The instrument used in all centers was a periodontal probe (PCP-UNC 15, Hu-Friedy, Chicago, IL, USA).

#### 4.4.2. Microbiological Assessment

The selected tooth was isolated with cotton rolls and gently dried with air. A sterile paper point ISO 45 (Inline, Torino, Italy) was inserted for 1 min in the residual pocket, placed into sterile Eppendorf tubes containing 200 micro liters of DMSO (Sigma Aldrich, St. Louis, Missouri, United States) and 15 glass beads (about 100 mg Bio-spec products) (Bartesville, Oklahoma, USA), and stored at −20 to −80 °C until laboratory analysis. The PCR tests of all centers were sent to Molecular Biology Service, Azienda Ospedaliera Universitaria, Cagliari, Italy. The total bacterial count and presence of specific bacterial species (*Porphyromonas gingivalis*, *Treponema denticola*, *Tannerella forsythia*, *Fusobacterium nucleatum*) were investigated.

### 4.5. Statistical Analysis

Numerical variables were reported as median (IQR) while categorical variables were reported as frequency (percentage). The McNemar test was used to test for differences in proportions between time points within groups and the chi-squared test was used for differences in proportions between groups. For numerical variables, time-points differences were assessed with the Kruskal–Wallis test and pairwise comparisons. Holms correction was used to adjust for multiple comparisons. A significance level of 0.05 was used for all statistical tests. All the analytical tests were performed using the statistical software R (version 3.6.0).

## 5. Conclusions

This is the first study to provide data on the effect of HA on residual pockets. Within its limitations, the present results conclude that the use of HA in adjunction to SI in the treatment of residual periodontal pockets did provide a better clinical and microbiological improvement than placebo in adjunction to SI in Step 3 therapy, but the improvement did.

## Figures and Tables

**Table 1:** Clinical parameters and differences between groups and between different recalls.

	T0	*p* Value _0–3_	T3	*p* Value _3–6_	T6	*p* Value _6–9_	T9	*p* Value _9–12_	T12
PD1	6 (5–7)	<0.001 *	5 (4–5)	<0.001 *	4 (3–5)	0.4	4 (3–5)	0.4	4 (3–5)
PD2	6 (5–7)	<0.001 *	5 (4–6)	0.001 *	4 (3–5)	0.5	4 (3–5)	0.003 *	4 (3–4.5)
*p* value	1.0		1.0		1.0		1.0		1.000
CAL1	7 (6–8)	<0.001 *	5 (4–6.7)	<0.001 *	5 (3–6)	1.0	5 (3–6)	1.0	4 (3–6)
CAL2	6 (5–8)	<0.001 *	5 (4–6)	0.002 *	5 (3–6)	0.5	5 (3–6)	0.07	5 (3–5.5)
*p* value	1.0		1.0		1.0		1.0		1.000
BoP1	48 (77.4%)	<0.001 *	33 (53.2%)	1.0	25 (40–3%)	1.0	19 (31.1%)	1.0	23 (37.7%)
BoP2	43 (67.2%)	<0.001 *	33 (51.6%)	0.8	30 (46.9%)	0.8	16 (25.4%)	0.8	15 (23.8%)
*p* value	1.0		1.0		1.000		1.0		0.690
Plaque1	26 (41.9%)	<0.001 *	21 (33.9%)	0.2	14 (22.6%)	1.0	21 (34.4%)	1.0	16 (26.2%)
Plaque2	18 (28.1%)	<0.001 *	14 (21.9%)	0.2	14 (21.9%)	0.5	23 (36.5%)	0.2	20 (31.7%)
*p* value	0.8		0.8		1.0		1.0		1.000
PD ≥ 5 mm1	64 (100%)	<0.001 *	38 (59.4%)	0.01 *	29 (45.3%)	1.0	25 (39.7%)	1.0	16 (25.4%)
PD ≥ 5 mm2	62 (100%)	<0.001 *	32 (51.6%)	0.4	22 (35.5%)	1.0	20 (33.3%)	1.0	17 (27.9%)
*p* value	1.0		1.0		1.0		1.0		1.0

The clinical parameters measured from the HA group are encoded with number 1, while the clinical parameters from the placebo group are encoded with number 2. The different recall visits are as follows: T0 (baseline), T3 (3 months), T6 (6 months), T9 (9 months) and T12 (12 months). PD (probing depth) is expressed as median and interquartile range CAL (clinical attachment level) is expressed as median and interquartile range; BoP (bleeding on probing) is expressed as the number of sites positive with bleeding on probing and as a percentage; plaque is expressed as the number of sites with plaque and as a percentage; residual pockets (PD ≥ 5 mm) are reported as the number of sites with probing depth equal and higher than 5 mm. *p* values are reported between treatment groups and between adjacent time recalls. * The statistical significance was set at 5%.

**Table 2:** Percentage of completely closed pockets (PPD ≤ 3 mm), shallow pockets (PPD ≤ 4 mm) and percentage of shallow pockets with lack of bleeding on probing (BOP) achieved in both treatments based on the values of PPD at T0 (baseline).

PPD0 = 5 mm					
		**T3**	**T6**	**T9**	**T12**
PPD ≤ 3 mm	Ha	53% (9/17)	65% (11/17)	65% (11/17)	56% (9/16)
	Placebo	27% (6/22)	50% (11/22)	55% (12/22)	59% (13/22)
	*p* value	0.1	0.4	0.5	0.9
PPD ≤ 4 mm	Ha	71% (12/17)	82%(14/17)	76% (13/17)	81% (13/16)
	Placebo	55% (12/22)	73% (16/22)	86% (19/22)	86% (19/22)
	*p* value	0.3	0.5	0.4	0.7
PPD ≤ 4 mm without BOP	Ha	59% (10/17)	71% (12/17)	76% (13/17)	69% (11/16)
	Placebo	55% (12/22)	59% (13/22)	82% (18/22)	82% (18/22)
	*p* value	0.8	0.5	0.7	0.4
**PPD0 ≥ 6 mm**					
PPD ≤ 3 mm	Ha	13% (6/45)	29% (13/45)	35% (15/43)	44% (19/43)
	Placebo	19% (8/42)	21% (9/42)	24% (10/41)	44% (18/41)
	*p* value	0.5	0.4	0.3	1
PPD ≤ 4 mm	Ha	40% (18/45)	58% (26/45)	60% (26/43)	70% (30/43)
	Placebo	33% (14/42)	45% (19/42)	46% (19/41)	68% (28/41)
	*p* value	0.5	0.2	0.3	0.9
PPD ≤ 4 mm without BOP	Ha	33% (15/45)	53% (24/45)	51% (22/43)	58% (25/43)
	Placebo	24% (10/42)	29% (12/42)	37% (15/41)	61% (25/41)
	*p* value	0.3	0.02 *	0.2	0.8
**PPD0 Total**					
PPD ≤ 3 mm	Ha	24% (15/62)	39% (24/62)	43% (26/60)	47% (28/60)
	Placebo	22% (14/64)	31% (20/64)	35% (22/63)	49% (31/63)
	*p* value	0.8	0.4	0.3	0.8
PPD ≤ 4 mm	Ha	46% (30/62)	89% (40/62)	69% (39/60)	77% (43/60)
	Placebo	40% (26/64)	54% (35/64)	62% (38/63)	78% (47/63)
	*p* value	0.8	0.5	0.1	0.6
PPD ≤ 4 mm without BOP	Ha	40% (25/62)	58% (36/62)	59% (35/60)	59% (36/60)
	Placebo	35% (22/64)	37% (25/64)	54% (33/63)	68% (43/63)
	*p* value	0.4	0.2	0.7	0.5

* p values for the difference between treatment groups are reported and considered significant at 5%.

**Table 3:** Microbiological parameters and differences between groups and between different recalls.

Table Title	T0	*p* Value _0–3_	T3	*p* Value _3–6_	T6	*p* Value _6–9_	T9	*p* Value _9–12_	T12
TBC1	11,850 (3200–53,690)	0.004 *	4130 (326–42,865)	0.2	2539 (468–7922)	1.0	2882 (137–11,500)	1.0	4626 (575–15,217)
TBC2	9813 (2917–54,970)	0.001 *	2125 (175–43,572)	0.05	2670 (1000–8995)	0.6	1700 (213–10,295)	0.05	8335 (330–32,700)
*p* value	1.0		1.0		1.0		1.0		1.0
Pg1	0 (0–4.1)	0.03 *	0 (0–0)	1.0	0 (0–0)	1.0	0 (0–0)	1.0	0 (0–0)
Pg2	0 (0–0.5)	0.1	0 (0–0)	1.0	0 (0–0)	1.0	0 (0–0)	1.0	0 (0–0)
*p* value	1.0		1.0		1.0		1.0		1.0
Td1	0 (0–0)	1.0	0 (0–0)	1.0	0 (0–0)	1.0	0 (0–0)	0.1	0 (0–0)
Td2	0 (0–0)	1.0	0 (0–0)	1.0	0 (0–0)	1.0	0 (0–0)	1.0	0 (0–0)
*p* value	1.0		0.6		1.0		1.0		1.0
Tf1	0.1 (0–6.6)	0.6	0 (0–3.6)	1.0	0 (0–0)	1.0	0 (0–3.7)	1.0	0 (0–0.4)
Tf2	0.2 (0–7.7)	0.3	0 (0–0)	1.0	0 (0–0.1)	1.0	0 (0–0)	1.0	0 (0–2.3)
*p* value	1.0		0.6		1.0		0.05		1.0
Fn1	5 (0.1–40.5)	1.0	3.01 (0–36.9)	0.04 *	30.9 (0.04–63.1)	0.01	5.1 (0.4–24)	1.0	10.3 (0.6–35.6)
Fn2	4.95 (0.1–24.7)	1.0	7.85 (0–50.8)	1.0	18.9 (1.1–55)	0.4	7.9 (0.9–26.6)	1.0	20.6 (0.6–46.3)
*p* value	1.0		1.0		1.0		1.0		1.0

The microbiological parameters measured from the HA group are encoded with number 1, while those from the placebo group are encoded with number 2. The different recall visits are as follows: T0 (baseline), T3 (3 months), T6 (6 months), T9 (9 months) and T12 (12 months). TBC, total bacterial count; *Pg*, *Porphyromonas gingivalis*; *Td*, *Treponema denticola*; *Tf*, *Tannerella forsythia*; *Fn*, *Fusobacterium nucleatum*. * The statistical significance was set at 5%.

## Data Availability

Not applicable.
